# Quality improvement practices to institutionalize supply chain best practices for iCCM: Evidence from Rwanda and Malawi

**DOI:** 10.1016/j.sapharm.2016.07.003

**Published:** 2017-11

**Authors:** Yasmin Chandani, Malia Duffy, Barbara Lamphere, Megan Noel, Alexis Heaton, Sarah Andersson

**Affiliations:** a JSI Research & Training Institute, Inc., Ground Floor Acacia Building, Westlands Office Park, Westlands, Nairobi, Kenya; b JSI Research & Training Institute, Inc., 1616 Fort Myer Dr. Arlington, VA 22209, USA; c John Snow, Inc. 44 Farnsworth Street, Boston, MA 02210-1211, USA

**Keywords:** Integrated community case management, Supply chain management, Community health workers, CHW, community health worker, iCCM, integrated community case management, QI, quality improvement, SMS, short messaging service

## Abstract

**Background:**

Supply chain bottlenecks that prevent community health workers (CHWs) from accessing essential medicines significantly increase under-5 child mortality, particularly in poor and rural areas.

**Objective:**

Using implementation research, interventions aimed at improving supply chain practices and access to medicines were tested in Malawi and Rwanda. These interventions included simple demand-based resupply procedures, using mobile technology and traditional methods for communication, and multilevel, performance-driven quality improvement (QI) teams.

**Methods:**

Mixed-method evaluations were conducted at baseline (2010), midline (2013), and endline (2014). Baseline assessments identified common bottlenecks and established performance levels. Midline assessments identified which intervention package had the greatest impact. Endline surveys measured the progress of scale-up and institutionalization of each innovation.

**Results:**

In both Rwanda and Malawi CHWs, health center staff, and district managers all cited many benefits of the establishment of resupply procedures and QI teams: such as providing structure and processes, a means to analyze and discuss problems and enhance collaboration between staff.

**Conclusions:**

Implementing simple, streamlined, demand-based resupply procedures formed the basis for informed and regular resupply, and increased the visibility of appropriate and timely community logistics data. QI teams played a critical role in reinforcing resupply procedures and routinely unlocking the bottlenecks that prevent the continuous flow of critical health products. While simple, streamlined, demand-based resupply procedures provide the basis for regular, functional, and efficient resupply of CHWs, the procedures alone are not sufficient to create consistent change in product availability. Supporting these procedures with multilevel QI teams reinforces the correct and consistent use of resupply procedures.

## Introduction

With mortality rates of 42 out of 1000 and 64 out of 1000 for children under 5 years of age in Rwanda and Malawi respectively, supply chain bottlenecks that prevent community health workers (CHWs) from accessing essential medicines significantly increase children's vulnerability, particularly in poor and rural areas.^1^, ^2^ Interventions, such as antibiotics for pneumonia, oral rehydration therapy and zinc for diarrhea, and antimalarials for malaria, have proven to reduce under-5 mortality by as much as 60%.^2^ Despite the evidence-base for such interventions, there is a dearth of literature on causes of supply chain bottlenecks or interventions to improve supply chain access.^3^ Identification of the root causes of supply chain barriers may contribute to enhancing the effectiveness of CHWs and improving under-5 mortality rates.^2^

The Supply Chains for Community Case Management Project (SC4CCM), funded by the Bill & Melinda Gates Foundation, was a learning project (2009–2015) that worked to identify proven, simple, affordable solutions to address supply chain challenges affecting CHWs. Working with government integrated community case management (iCCM) programs in Malawi and Rwanda, the project identified bottlenecks and tested interventions, through implementation research, aimed at improving supply chain practices and access to medicines so CHWs can better treat common childhood illnesses.

The limited research that exists primarily focuses on identifying and addressing malaria treatment barriers. A study by O'Connell and colleagues that examined antimalarial stock availability in 6 sub Saharan African countries found that antimalarial availability for CHWs ranged between 26% and 80%. The authors did not identify product availability bottlenecks.^4^ Mobile phone technology is increasingly recognized as a means to address product availability.^5^, ^6^, ^7^ The SMS for Life pilot in Tanzania tested the impact of health worker SMS use via mobile phones to provide information on stock counts for district management teams. A reduction in stockouts of one or more antimalarials in pilot health centers from 78% to 26% was noted throughout the 21 week study.^6^ Blanas and colleagues examined the effectiveness of trainings for lay health workers in implementing new malaria community case management protocols. Despite post-training knowledge and skill improvements, stockouts of rapid tests and antimalarials occurred in half of the pilot communities, significantly impairing lay health workers' capacity to provide malaria services. The authors likewise did not identify barriers to product availability.^8^

Studies that examined challenges for CHWs to implement iCCM identify supply chain as a significant barrier. A study examining barriers to iCCM in South Sudan, Uganda, and Zambia found that stockouts were common across all 3 countries and often occurred during rainy season. The lack of available medication severely limited the capacity of CHWs, led to poor community attitudes toward iCCM and CHWs in general, and resulted in increased attendance at health centers.^9^ A survey that examined availability and cost of essential medicines in 14 sub-Saharan African countries found that only 3 countries had Central Medical Stores with greater than 50% of national essential medications available.^10^ An OxFam study in Malawi found that only about 9% of health centers had sufficient stocks of essential medicines, including for pneumonia and diarrhea, requiring ill patients to be treated with inappropriate medication, travel further distances to reach health centers with the appropriate medicine, visit a private and more expensive health care provider, visit a traditional healer, or go without treatment.^11^, ^12^

There were no studies identified that used quality improvement (QI) to address supply chain barriers. However, there are compelling results from studies that use QI approaches to address other challenges that CHWs encounter. A study in rural Uganda that used QI methods to address routine immunization gaps found that the approach provided a framework to use local solutions and significantly improved the reach of routine immunization with changes sustained 5 months after the intervention.^13^ The Kabeho Mwana project in Rwanda used QI approaches among CHWs to improve iCCM care seeking behaviors. Study findings indicated that care seeking for fever, diarrhea, and acute respiratory infection significantly increased in intervention districts.^14^ In Ethiopia, QI teams, including community stakeholders, traditional birth attendants, and health extension workers, identified birth notification gaps and tested interventions to increase postpartum visits by health extension workers within 48 h of birth. These interventions resulted in significant improvements in postnatal care access.^15^

Resupply procedures were developed for this study, including a paper-based resupply calculator in Rwanda and an electronic reporting and resupply system (cStock) in Malawi, to improve supply chain reporting and resupply. To compliment the resupply procedures, multilevel, multidisciplinary QI teams were established to reinforce the correct and consistent use of resupply procedures and tools and to monitor supply chain performance. Techniques were used to empower QI teams to use logistics data to take action to address supply chain problems and bottlenecks; creating a culture of shared responsibility and problem solving led the teams to expect a continuous supply of products, investigate when there were supply challenges, and participate in finding solutions. The project hypothesized that simple, demand-based resupply procedures, using mobile technology and traditional methods for communication, alongside multilevel, performance-driven QI teams, have the potential to improve long-term supply chain outcomes for CHWs.

### Rwanda

In Rwanda in 2010, the project collaborated with other stakeholders to develop and pilot a set of resupply procedures. From 2012 to 2013, the project tested a proven QI method to strengthen use of the resupply procedures to improve community supply chain performance and product availability for CHWs. The midline assessment found that both the resupply procedures and QI interventions were well-implemented and the QI teams at health centers met regularly. The QI process was considered valuable as a means for improved coordination and problem solving across levels of the health system.^3^, ^13^, ^14^, ^15^

Following the midline assessment, the resupply procedures and QI team intervention package was refined and the project provided support to the Ministry of Health (MOH) to scale it up to all districts in Rwanda. The goal of the standard resupply procedures was to keep the system as simple as possible while ensuring that CHWs always have enough products to serve their clients. This system assigns the lead CHW, called a cell coordinator, the responsibility for collecting and reporting logistics data and ensuring supplies reach all CHWs in their cell. To meet these objectives, requirements were condensed so that only three simple tools were needed for the CHW resupply process: a stock card, the resupply worksheet, and the magic calculator developed by the project. In this scale-up phase, the resupply procedures were added to an integrated training for cell coordinators which included other areas of iCCM training. The integrated training has been supported by various implementation partners in different districts. The training was cascaded from a team of Master Trainers to district and health center staff and then to cell coordinators to implement within their cells.

The scale-up intervention also included the same problem solving QI team approach that intended to reinforce the correct and consistent use of resupply procedures by creating QI teams that linked cell coordinators, health center, and district staff, as well as addressing any CCM product shortages and stockouts for the 5 key CCM products (amoxicillin 125 mg, zinc 10 mg, ORS, pediatric ACTs – Primo Red and Primo Yellow, and RDTs). The QI teams met monthly at the health center to compile and analyze data, identify problems, and develop or refine solutions that they thought would improve use of resupply procedures. Between meetings, the QI team members visited CHWs to implement the interventions agreed to at the QI team meetings through integrated supportive supervision activities.

In the original districts, during the intervention period, cell coordinators were provided allowances to facilitate the collection of QI data during supportive supervision visits to be used during QI team meetings. These allowances were discontinued and not included in the scale-up package. However, supervision visits were expected to continue as part of cell coordinator duties, using an integrated supervision checklist that included supply chain indicators as well as other programmatic indicators.

### Malawi

In Malawi, the Enhanced Management intervention was developed which consisted of health center and district level QI teams to oversee implementation of cStock, an SMS and web-accessible, open-source logistics management information system. cStock is used for reporting logistics data, calculating resupply, and managing and monitoring all community-level health products. cStock enables Malawi's CHWs to use their own mobile phones to transmit monthly stock level data and receipts to a toll-free short code, with no additional hardware costs or financial incentives. cStock automatically calculates resupply quantities from reported data, and sends reorder quantities to supervisors at health centers, facilitating accuracy and reducing transport time and costs. cStock generates reports on more than 10 supply chain indicators that program managers and partners can use for performance monitoring and supervision. At the district level and above, managers have access to a web-based dashboard to monitor performance. cStock was designed to mimic the paper reporting and resupply system in Rwanda.

The QI teams were formed with a shared vision and commitment to enhance proactive leadership in monitoring and managing product availability at district and health center levels. The teams met quarterly at the district level and monthly at the health center level, shared goals specifically around product availability and supply chain performance, used cStock data to assess progress against established team performance targets, addressed bottlenecks and challenges through problem solving, developed action plans to improve on weak areas, and recognized and rewarded outstanding individual performance within teams as motivation toward performance excellence. A midline assessment of the original QI teams and cStock intervention found that product availability at the community level had more than doubled from baseline to midline, and that the increase was driven partially by more products in the system, as well as the improvements in the supply chain system.^16^ Similar to Rwanda, following midline, the project assisted the MOH and partners to refine and scale up the interventions to all 29 districts in Malawi.

### Objective

Using findings from the endline assessments, this paper will discuss the results of scaling proven, simple demand-based resupply procedures, using mobile technology and traditional methods for communication, and establishing multilevel, performance-driven QI teams in Malawi and Rwanda, and the potential contributions these interventions had on supply chain outcomes for CHWs.

## Material and methods

**Fig. 1:**
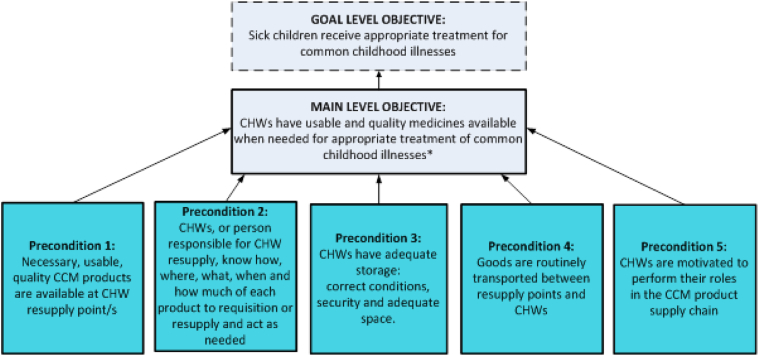
Five preconditions of community level product availability.

A Theory of Change (TOC) was developed as the guiding technical activity planning and evaluation framework. The TOC consists of a series of critical thinking exercises that examine early and intermediate-term changes required to meet a long-term goal that is specified by a community.^17^ The TOC was created through a literature review and multiple discussions with internal experts and field staff, and validated by an external group of international supply chain and evaluation experts. Using baseline data, country specific TOCs were developed that outlined key preconditions to community case management product availability for CHWs (Fig. 1).^3^

The baseline assessment, conducted in Rwanda and Malawi in 2010, identified common bottlenecks for medications to reach communities. Each country study took place in a total of 10 districts: 3 districts (Intervention 1), 3 districts (Intervention 2), and 4 districts (comparison, non-intervention districts). Districts were matched to create comparable intervention groups based on demographic, geographic, disease burden, and implementing partner profiles. Midline assessments were conducted in 2013 to identify which original intervention package had the greatest impact on improving supply chain performance at the community level, and to compare intervention districts against the 4 non-intervention districts. Findings from the midline assessments are detailed in separate publications.^3^, ^18^, ^20^ Following the midline assessment, the original interventions identified as improving product availability were refined based on findings and scaled up. Endline surveys, which took place in 2014, measured the progress of scale-up and institutionalization of each innovation and its contribution to improved supply chain practices.

A mixed-method approach was used for the endline surveys; qualitative data was collected using a case study methodology, and quantitative data was collected with a modified Logistics Indicator Assessment Tool in Rwanda and with cStock in Malawi.^21^ Qualitative data specifically explored how resupply procedures plus QI approaches were used, and how the approaches may or may not facilitate CHWs and cell coordinators to improve supply chain practices, focusing on the influence of the contextual and mediating factors and the relationship between these factors. Quantitative data provided specific information on the use of the paper-based system in Rwanda, cStock in Malawi, and product availability, visibility, and use of product availability for decision making in both countries. Endline findings explored the extent (geographic breadth and institutional depth) of the interventions when scaled up, the extent that observed program effects at midline were sustained to endline, and the institutionalization of the interventions. The endline also identified aspects of the intervention design, implementation, and overall project approach that contributed to the scalability and sustainability of the intervention.^16^, ^21^ The endline study design is described in Table 1.

**Table 1:** Overview of endline study design by country

Country	Sampling method	Selection criteria	Data collection	Analysis
Rwanda April–June 2014	**Case study**			
	− Purposive sampling of 2 health centers in 2 baseline districts	− Two original districts were purposively selected based on performance: highest and lowest change in product availability from baseline to midline	− 53 in-depth interviews with cell coordinators, CHW supervisors, health center pharmacy managers, district CHW supervisor, and district pharmacist	− Interview transcripts and observations were analyzed during workshop to identify emerging themes
	− Purposive sampling of a third baseline health center	− Two new districts were purposively selected based on where RSP scale-up had started, QI teams addendum training conducted, the partner support model, and having a health center profile similar to the original districts	− 52 observation, demonstration, collective discussions, or photos	− Second stage analysis compared original and scale-up districts, identifying institutionalization and formulating lessons from scale-up
	− Purposive sampling of 2 health centers in 2 scale-up districts			− Specific themes identified during the workshop were analyzed by different team members and findings were reviewed by the entire team
	**LIAT**			
	− Random sampling of 30 health centers to match baseline and midline numbers	− Randomly selected from QI teams districts	− 64 structured interviews with cell coordinator and 96 CHW structured interviews	− Chi-square using Stata SE 13
			− Measurement of tool availability, key activity status, information quality, and product availability	
Malawi June–July 2014	**Case study**			
	− Purposive sampling of 2 health centers in 2 baseline districts	− Two original districts were purposively selected based on performance at midline	− 24 in-depth interviews with CHWs	− Interview transcripts and observations were analyzed during workshop to identify emerging themes
	− Purposive sampling of 2 health centers in 2 scale-up districts	− Two new districts were purposively selected; 1 district where cStock was introduced before midline and QI teams were introduced after midline and 1 district where both cStock plus QI teams were introduced after midline	− 16 CHW interviews	− Second stage analysis compared original and scale-up districts; identifying institutionalization and formulating lessons from scale-up
		− Health centers were purposively selected based on performance (high reporting rates, low and high meeting occurrence)	− 15 interviews with program coordinators, district health officers or district medical officers, and pharmacy technicians.	− Specific themes identified during the workshop were re-analyzed by different team members and findings reviewed by entire team
			− 22 observations, demonstrations, collective discussions, photos	
	**cStock**			
	− All available data in cStock	− All CHWs using cStock nationally, plus district level data for case study districts	− Data was extracted for stock status, stockouts, reporting rate, order fill rate, and emergency orders for period between midline and endline (January 2013 –– May 2014)	− Trend analysis

### Case study analysis

In each country, transcripts from the case study interviews and observations were first analyzed during a 5 day in-country analysis workshop to identify emerging themes. Next, important contextual and mediating factors were identified, level by level, and incorporated into the analysis. The second stage analysis compared findings from original and scale-up districts, identifying evidence of institutionalization and formulating lessons from scale-up before developing the key findings statements. Different team members re-analyzed the specific themes and topics that were identified during the in-country analysis workshops, and the entire team reviewed the findings. Findings from the quantitative survey also informed further in-depth exploration of the case study data in Rwanda. Case study and quantitative results were then triangulated to identify concordance, discordance, and explanatory relationships. When concordance among findings between the 2 sources of data was found, the 2 sources of data were used to reinforce findings, and to provide more in-depth information on the processes and experiences of users of resupply procedures and participants in QI teams.

### Quantitative analysis

In Rwanda, lower level staff interviews were translated into Kinyarwanda. Both the training of enumerators and the data collection itself were carried out in English and Kinyarwanda as needed. Four teams composed of 2 enumerators each, plus 1 supervisor, traveled to field sites for data collection. Data were collected by enumerators using cell phones loaded with preset forms developed using Magpi software. Data on phones were stored in a password-protected web-based database that only researchers could access. Data collection forms and content were all Java-based, and were not sent over network phone lines, in SMS format in order to protect data. Quantitative data were analyzed using Stata SE 13. Chi-square tests were used to test the significance of results between midline and endline.

In Malawi, the cStock system was designed not only as a logistic management information system, but also as a tool for monitoring and evaluation, and as such, was designed to collect and visualize key supply chain indicator data over time. For the quantitative component of the endline evaluation, data for stock status, stockouts, reporting, order fill rate, and emergency orders were extracted from cStock to measure the performance of the community health supply chain between midline and endline (January 2013 – 2014). The use of routine cStock data for the evaluation allowed access to a much larger sample size of districts and CHWs, with a maximum sample size of 2512 available between February and May 2014. cStock allowed project staff to view supply chain trends by monthly intervals and compare them across pilot districts and more recent adopters. The analysis of cStock data facilitated a clearer and deeper understanding of whether the innovation effects on supply chain performance were sustained in the pilot testing districts, and whether new districts were beginning to demonstrate similar trends.

## Results

Midline results in Rwanda found that 63% of health workers in QI team districts had all 5 products in-stock compared to only 38% in districts without QI teams (*p* < 0.001). In Malawi, results showed significantly lower stockout rates with 5%–7% stockouts in districts with QI teams compared to 10%–21% in the districts with no QI team (*p* < 0.001). Full findings from the midline assessments are detailed in separate publications.^3^, ^18^, ^19^, ^20^

### Resupply procedures implementation

In Rwanda, all districts that were part of the case study or quantitative survey were found to have key tools for resupply procedures – stock cards, resupply worksheets (report and requisition), and the magic calculator – available and in use at endline. Case study observations showed that all 16 pairs of cell coordinators were able to correctly demonstrate the process used for completing the resupply worksheet. Cell coordinators, health center staff, and district staff, in both original and scale-up districts, identified a range of similar benefits that resulted from using the resupply procedures, such as providing organization and structure, clear responsibilities, ease of information, and transparency.

However, despite the knowledge and understanding of the benefits of resupply procedures, the endline evaluation in Rwanda found that cell coordinators and health center staff were not always using the procedures or tools correctly or consistently in both original and scale-up districts. In the quantitative survey, the proportion of health centers in the original districts that were able to show all resupply worksheets submitted in the previous month decreased from midline to endline, from 94% to 67%. A similar situation was observed in the case study; of the 16 pairs of cell coordinators observed, only 4 had 3 consecutive months of resupply worksheets available on the day of the study. The case study team found several instances of incomplete worksheets in their observations in both original and scale-up districts.

All CHWs that were visited in Malawi for the case study, from both original and scale-up districts, demonstrated a clear understanding of their reporting responsibilities; namely, CHWs knew they were to report stock on hand every month to cStock, to report receipts to cStock when they collected products, and to send an emergency order when stocks were low. Case study findings supported the quantitative data from cStock which showed that reporting rates for stock on hand reports in all 4 districts were consistently above 80%, and often greater than 90%, and were equal or above the national reporting rate. Common benefits of cStock that respondents cited included ease of reporting, time saved, reduced workload, and improved communication and transparency between CHWs and health center staff.

District and central level managers in Malawi reported the benefits of the cStock system in producing timely, usable data through the dashboard. Managers cited that they were able to easily monitor the system and better assess the needs of the lower levels, and that this complemented the QI structures.

*“With the dashboard, we can look on there to see what's going well and what's not, and we can contact people … we can call someone and say, ‘I've noted from the dashboard that you haven't done 1, 2, 3, 4, do you need any support?’ Or if they're doing well, we can also recognize them … What I saw in the [national level QI team] and [district QI team] is that it [the dashboard] makes us know what is going on, like the stock levels nationally. It just needs someone to have an interest in it, because programs need to look on cStock, and to do that, they have to have the interest. And when there are challenges at the different levels, at the national level, at the district or in the village clinics, each level has a role to play. When there is a problem or an accomplishment, everyone appreciates it.”* Central MOH, Malawi

### Adherence to QI processes

In Rwanda's scale-up package, QI data was collected through a newly introduced integrated supervision checklist that cell coordinators were supposed to use when visiting CHWs in their cells. Of the 3 original districts included in the quantitative survey, only 10% of cell coordinators could show integrated supervision checklists for all CHWs in their cell for the past quarter with the supply chain portion complete, implying limited data available for use in the QI team process and meetings. Of cell coordinators, 70% said they were trained on using the integrated supervision checklist, and 84% said they were using it; however, only 55% of cell coordinators could show at least 1 copy of a completed checklist on the day of visit.

Limited data from cell coordinator supervision visits reduces the impact of QI team meetings as the data from the supervision checklists is instrumental for the QI team to understand the range of supply chain issues and prioritize the most important problems to address. Quantitative survey data from the original districts show that of the 30 health centers surveyed, 27 reported having at least one QI team meeting in the last year and the majority had only had one meeting. Twenty-one health centers had held a QI meeting in the last quarter, of which 16 health centers had documentation of that meeting. Of those 16 health centers who had documentation of holding a QI team meeting, only 13 (81%) could show the expected QI team tools completed for that meeting (action plan with SMART objective). Similarly, while the majority of cell coordinators (78%) reported attending a QI team in the previous 3 months, meeting documentation for the QI team was observed for only 53% of meetings.

In Malawi, QI activities at the health center level were typically conducted around resupply procedures. Six out of the 8 surveyed health centers had monthly QI team meetings. In 1 health center in an original pilot district, QI meetings had stopped in 2013 because of a staff change. At 1 health center in a scale-up (new) district, motivation to continue meetings dwindled after 2 meetings due to the impression that the district was not responding to the team's requests that they identified would help solve product availability problems; one request consisted of procuring supply bags and rain gear to address transport related challenges, for which the district did not have funds.

All health centers had management diaries, but none had copies of the district performance plan or recognition plan. Based on observations from the management diaries, most QI team meetings focused on the recommended performance indicators: timely and complete reporting, issues related to lead time, stock on hand, emergency orders – different aspects of supply chain monitoring that need to occur routinely (Table 2). Non-reporting and lack of timely and complete reporting were the most frequently discussed issues in both original and scale-up districts, followed by lead time and specific product stock issues.

**Table 2:** Malawi performance monitoring by district and health center

	Original district				Scale-up districts			
	District A		District B		District C		District D	
	HF 1	HF 2	HF 1	HF 2	HF 1	HF 2	HF 1	HF 2
Document used for monitoring	No mention	Report from district	No mention	Resupply worksheet	Resupply worksheet	Resupply worksheet	Form 1A	No mention
Performance plan	No	No	No	No	No	No	No	No
Management diary	Yes, not observed	Yes*	Yes	Yes	Yes	Yes	Yes	Yes
Recognition plan	No	No	No	No	No	No	No	No
Indicators discussed during meetings**								
Reporting	n/a	X	X	X	X	X	X	X
Lead times	n/a	X	X		X	X	X	
Emergency orders	n/a	X		X	X			
Stock issues	n/a	X			X		X	X

*Observed management diary entries only from 2011 to August 2013 when meetings were still occurring; **Management diaries observed from November 2013 to June 2014.

### Benefits of QI approach

In both countries, CHWs, health center staff, and district managers cited many benefits of the establishment of QI teams and meetings. It was recognized that QI activities provided a means to analyze and focus on CHW challenges, assisted cell coordinators to identify problems in their cells, made practical use of supervision data, allowed for discussion of problems and collaboration with multilevel staff, specified regular times for cell coordinators to speak to the supply chain challenges within the cell, and established a timeline and accountability for problem solving. Other benefits of the QI teams were related to improved relationships and CHW engagement with their work and colleagues, which are difficult to measure but affect motivation and attention to overall job performance. As CHWs are often volunteers and/or working in remote locations, and are loosely associated with the formal health system, these “soft” benefits should not be undervalued. Formalizing relationships and establishing teams with common objectives can make CHWs feel more valued, and positively impact their job satisfaction and ability to do what is needed to meet their individual job expectations. These benefits are summarized in Table 3 below and illustrated by quotations that represented a common opinion of QI team members in the qualitative endline data.

**Table 3:** Perceived benefits of QI team meetings

Perceived benefit	Key quotations
Enables collective problem solving	*“With [QI] meetings I am now able to ask friends if I have problems.”* CHW, Malawi
	*“The health center Data Manager, the CHW Supervisor, and I meet with the cell coordinators monthly. We ask the cell coordinators to bring the tools that they use, like the resupply worksheet, the tally sheet, and the integrated supervision checklist. We analyze data, identify problems together, and try to come up with a work plan.”* Health center pharmacist, Rwanda
Facilitates better performance	*“You correct the mistakes that you were making, be it reporting late or coming late to collect products when advised to do so.”* CHW, Malawi
	*“QI team meetings are very important because they help us to discuss all problems we identified during supervision and find quick solutions to them and determine the timeline for actions we took, identify who is responsible of this action and evaluate its implementation status during the next meeting.”* CHW, Rwanda
Enables knowledge and experience sharing	*“A lot because it has improved the way I am working considering that there are times where I am able to meet and share experience with colleagues.”* CHW, Malawi
	*“QI team meeting is good, it helps us working together and we help each other and we discuss on how we can improve our work, and what you don't understand you ask your colleague to orient you how to do it.”* CHW, Rwanda
Allows mutual encouragement and motivation and improved relationships	*“We encourage each other to work harder on cStock, e.g. telling each other the importance of sending our different reports at the right time.”* CHW, Malawi
	*“Also the QI team meetings have helped to improve our relationship with the health center staff (especially Pharmacy Manager and CHW Supervisor), we know whom to talk to at the health center depending on the problems we have.”* CHW, Rwanda
Increases coordination and collaboration between levels of the health system	*“ ….but now we are meeting frequently and there is coordination among us …”* CHW, Malawi
	*“Collaboration between CHWs and cell coordinators were not happening before. With QI team meetings, we know the problems, the weaknesses, and the actions that need to take place. When I'm sitting next to cell coordinators and hear from them, I can really understand their problems. And if it's a problem related to supply chain, I can help resolve it. The meetings are really good … Oh, let me also mention that there is now collaboration between the district pharmacy and the community. Before, we only worked with the health centers. But now, we are aware that we need to interact with the community level. It's a big deal.”* District pharmacist, Rwanda

### Challenges of QI approach

In both countries, a main challenge to implementing the QI approach was the lack of district engagement. District staff were trained to provide leadership and oversight to the monthly QI team meetings at the health center. In Rwanda, the model was that district staff would hold an initial “kick-off” meeting to introduce the concepts; review the data, tools, and meeting structure; and help the team to schedule monthly meetings. After that, the district coach was to be available as a resource and attend a meeting of each QI team at least once per quarter. As the intervention scaled up, these kick-off meetings were limited by lack of district engagement – either because the district staff were busy with other competing activities, or often because there was no accountability or central level person asking them about the meetings. An additional challenge to implementing the QI approach was finding the resources and time required to travel and attend monthly meetings for busy health center staff and CHWs. These challenges are further described in Table 4.

**Table 4:** Perceived challenges related to regular monthly team meetings

Challenge	Key quotation
Transportation/distance	*“CHWs stay too far away so they can come late for meetings and to collect drugs. Sometimes they have to use their own money to get here. Some CHWs have bikes, some don't.”* Health center in-Charge, Malawi
	*“Ooh! You see we came from far for the QI team meeting and later after travel long distances to visiting CHWs in their villages. We do all this per volunteerism but it is really difficult for us. As we do not have time to perform our ordinary work, it would be better if we can have transport allowances to facilitate us to properly achieve our responsibilities.”* CHW, Rwanda
Length of meeting and lack of refreshments/allowances	*“No there are no problems – we have bicycles – but the main problem is having to spend a long time there without taking any food.”* CHW, Malawi
	*“The challenges are related to lack of means, you see we come and attend those meetings; we leave our homes while we would be working on other living activities in our families, maybe if we could get something to facilitate us in transport to attend in the meeting or a Fanta when we attend in that meeting it will be good.”* CHW, Rwanda
Other competing obligations	*“In June, the 1st was on a Sunday so the CHWs failed to come. Some came on Monday to get their products. Some came on Tuesday.”* CHW supervisor, Malawi
	*“Yes, QI team meetings were important because they helped us to identify problems and find solutions. Considering their importance, we wish they may continue, and if possible find from the central level how to provide allowances as motivation. For example, we travel for about 2 h to reach the health center, which means 4 h traveling to getting back home. So if it is a meeting day, all other activities are abandoned.”* CHW, Rwanda
Lack of district engagement and feedback	*“I think people from the district are lacking seriousness, of late they are not even asking us why we are not meeting.”* CHW, Malawi
	*“We tried to re-launch in February but were very busy with other activities. We've been talking about how to get the meetings started again. We have a plan to restart at the beginning of June. We have a meeting next week to discuss this issue.”* District pharmacist, Rwanda

### Supply chain outcomes for CHWs

The endline evaluation focused on assessing whether the product availability improvements were sustained in the original pilot districts compared to the midline results, and if scale-up districts had experienced changes in product availability since the intervention was introduced.

**Fig. 2:**
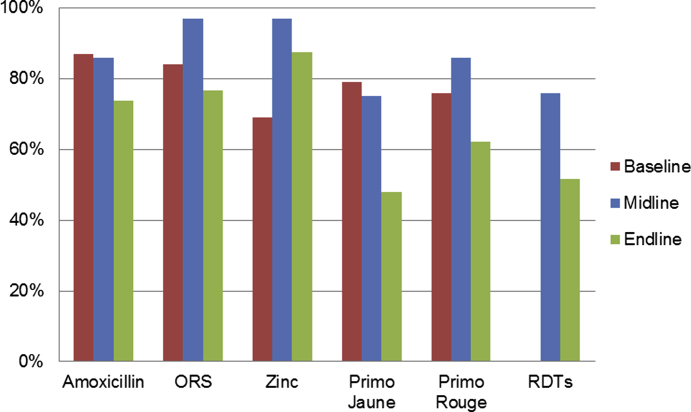
Percent of CHWs in-stock on day of visit.

In Rwanda, the midline assessment demonstrated the effectiveness of the interventions on improving product availability at the health center and cell coordinator levels. The focus for the endline evaluation was on whether the improvements in product availability were sustained in the original districts (Fig. 2); these results required careful interpretation since the intervention itself changed significantly based on the midline results and plans for scale-up. Data from the quantitative survey showed that availability of all 5 products among CHWs returned to baseline levels after reaching a peak 1 year earlier, when midline measurements were made in the original districts.

While the endline results may first appear as a failure to sustain initial pilot project effects, it is important to recognize that the intervention was substantially changed and not fully implemented between midline and endline, so that what was assessed at endline was in many ways a new, partially implemented intervention, even in the original districts. The most significant changes, in addition to a reduction in project inputs, included: 1) removal of allowances provided to cell coordinators and district coaches; 2) introduction of integrated training and supervision; and, 3) changes in both the magic calculator and the recommended QI team tools. The first change most likely had the greatest effect on product availability, since it directly affected the motivation of cell coordinators to conduct regular supervision and attend QI team meetings, and reduced the regularity of QI team meetings as district coaches were much less likely to attend. External factors that affected product availability at the national level likely had an impact as well, but it was not possible to control for or dissociate these with this endline design.

**Fig. 3:**
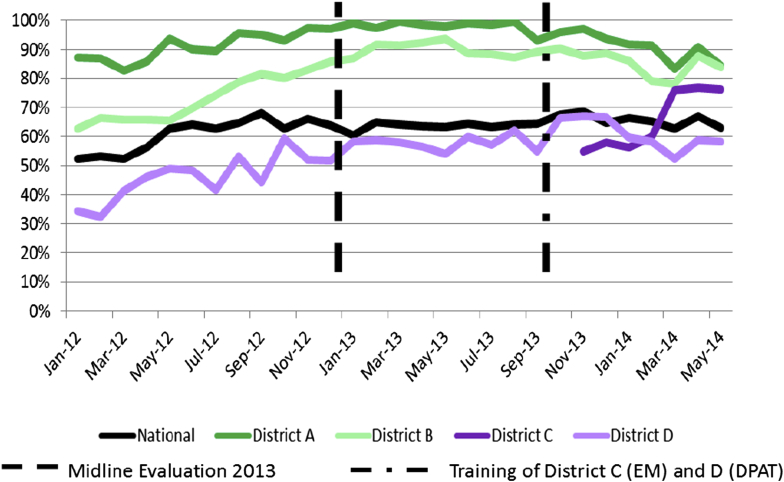
Average in-stock rates across 5 CCM products.

In Malawi, cStock data was analyzed for the period starting before midline (January 2012), when the intervention had been rolled out to the 6 pilot districts, until the endline (May 2014), when up to 24 districts had progressively been trained and had started to use cStock. Fig. 3 represents the average in-stock rates among CHWs across the 5 CCM products for the case selection districts and nationally. To calculate this figure, the percent of CHWs in-stock was calculated for each individual product, and then the average percent of CHWs in-stock was calculated for the 5 products.

## Discussion

Community level product availability is a measure of the performance of the entire supply chain, and as such, is influenced by numerous factors. Key prerequisites for product availability include: 1) having enough product available at the national level to supply all programs and levels; 2) a supply chain that functions effectively, consistently, and ensures continuous availability of products across all levels of the supply chain, including the health center – a common resupply point for CHWs; and 3) a standardized resupply process for the community level that aligns product and data flow with higher levels. The project's mandate was to remove barriers between the CHWs and resupply point, with limited influence over national product availability, except to support routine quantification using the better data visibility provided by the resupply procedures. The project found that establishing appropriate resupply procedures at the community level and implementing supportive teams that use QI approaches to collectively review data and problem solve created a more responsive supply chain, resulting in significant and measurable improvements in key supply chain processes and contributing to improved product availability.

In both Rwanda and Malawi, implementing simple, streamlined, demand-based resupply procedures formed the basis for informed and regular resupply, and increased the visibility of appropriate and timely community logistics data at both health center and district levels. With increased data visibility, managers and QI teams were able to regularly monitor supply chain performance and respond in a timely and targeted way. The SMS and web-based mHealth system in Malawi showed benefits over the paper-based reporting used in Rwanda, as the system quickly transformed and visualized data into relevant, aggregated reports that improved usability of community health logistics data at all levels of the supply chain. However, in both countries, it was clear that well-designed and implemented resupply procedures and improved data visibility were not sufficient on their own to create consistent change in product availability at the community level.

The research findings showed that QI teams play a critical role in routinely unlocking bottlenecks at district and health center levels that prevent the continuous flow of products between these levels. Teams promote a customer service-oriented mindset and a culture that enhances communication and collaboration, and prioritizes problem solving around – and a shared responsibility for – resolving product availability issues. In Malawi, the use of cStock for reporting was shown to be consistent across both original and scale-up districts in the endline evaluation. The QI team meetings provided a forum that facilitated continued use of the data provided through cStock for performance monitoring, tracking progress on goals and targets, and stock management. In Rwanda, the endline evaluation clearly confirmed that resupply procedure knowledge and skills alone were not enough to sustain correct and consistent use of the procedures at the community level, even if users recognized multiple, wide-ranging benefits. Continuous support and communication, facilitated and provided through the QI team, remained necessary.

In order to be effective, QI teams rely on a clear, replicable process for using data to identify performance targets, developing action plans toward meeting these performance targets, and then monitoring progress. In both countries, 3 elements proved to be critical for an effective QI approach:

1) Reliable data source: such as cStock dashboard, reports, and supervision checklists;
2) Easy to use tools: indicator tally sheet, management diary, why–why analysis, and action plan; and
3) Structured process: regular QI team meetings to review and use data to track progress against objectives and determine actions to improve performance.

Building on the midline evidence base, endline findings showed that QI approaches that address resupply procedures can be successfully adapted to improve supply chain performance by reinforcing processes, introducing a culture of problem solving, and improving teamwork and motivation, when there are key elements in place and sufficient management and leadership support. The endline evaluation in Rwanda found that there was appreciation and enthusiasm for the idea of having QI teams in both original and scale-up districts, but also confirmed that QI teams required initiative and motivation from the trained coaches at the district level to actually get them started. The case study data showed the removal of allowances after the pilot consistently cited by both cell coordinators and health centers as the primary barrier to QI teams; they discussed the demotivating, discouraging aspects, as well as how not receiving compensation for this work made it difficult to prioritize spending time on QI team duties and supervision over income generating activities.

The QI team mechanism was intended to mitigate imbalances in supply by improving communication and transparency between the levels responsible for ensuring product flow, and encouraging continued and consistent use of procedures while supply issues were being resolved. The evidence from comparing midline results with endline results suggests that when consistent QI team meetings were held, they played a role in ensuring consistent and continued use of resupply procedures by facilitating communication, transparency, and a sense of accountability when supply issues arose. However, case study findings suggest that in the absence of regular QI team meetings, while resupply procedures continue to be seen as the primary mechanism for resupply, the tools and processes are followed less consistently and correctly, which in turn affects overall product availability.

## Conclusion

Study findings demonstrate that implementing simple, streamlined, demand-based resupply procedures, a known best practice in supply chain, provides the basis for regular, functional, and efficient resupply of CHWs, but is not sufficient to create consistent change in product availability. Supporting these procedures with multilevel QI teams that improve communication and coordination between staff at different levels reinforces the correct and consistent use of resupply procedures, and facilitates monitoring of supply chain performance and action planning to address supply chain problems and bottlenecks. The combination of well-designed resupply procedures with the establishment of a QI team resulted in significant and measurable improvements in key supply chain processes and contributed to improved product availability. However, reliable availability of health commodities at the community level is not possible without dependable national level availability and a higher level supply chain that facilitates efficient movement of community products to resupply points and data to and from all levels of the system.

## Author contribution

All authors contributed equally to this work.
